# Electrically Switchable Polymer Brushes for Protein Capture and Release in Biological Environments**

**DOI:** 10.1002/anie.202115745

**Published:** 2022-03-30

**Authors:** Gustav Ferrand‐Drake del Castillo, Maria Kyriakidou, Zeynep Adali, Kunli Xiong, Rebekah L. N. Hailes, Andreas Dahlin

**Affiliations:** ^1^ Department of Chemistry and Chemical Engineering Chalmers University of Technology Kemigården 4 41296 Göteborg Sweden

**Keywords:** Adsorption, Electrochemistry, Polymer Brushes, Proteins

## Abstract

Interfaces functionalized with polymers are known for providing excellent resistance towards biomolecular adsorption and for their ability to bind high amounts of protein while preserving their structure. However, making an interface that switches between these two states has proven challenging and concepts to date rely on changes in the physiochemical environment, which is static in biological systems. Here we present the first interface that can be electrically switched between a high‐capacity (>1 μg cm^−2^) multilayer protein binding state and a completely non‐fouling state (no detectable adsorption). Switching is possible over multiple cycles without any regeneration. Importantly, switching works even when the interface is in direct contact with biological fluids and a buffered environment. The technology offers many applications such as zero fouling on demand, patterning or separation of proteins as well as controlled release of biologics in a physiological environment, showing high potential for future drug delivery in vivo.

## Graphical abstract

pH‐responsive polymer brushes are presented that are compatible with electrochemical control. The brushes can be reversibly switched between protein binding and repelling states at fully physiological conditions with respect to salt, pH and buffering capacity. Proof‐of‐principle experiments are shown for applications such as non‐fouling, patterning and controlled release of proteins in biological environments.

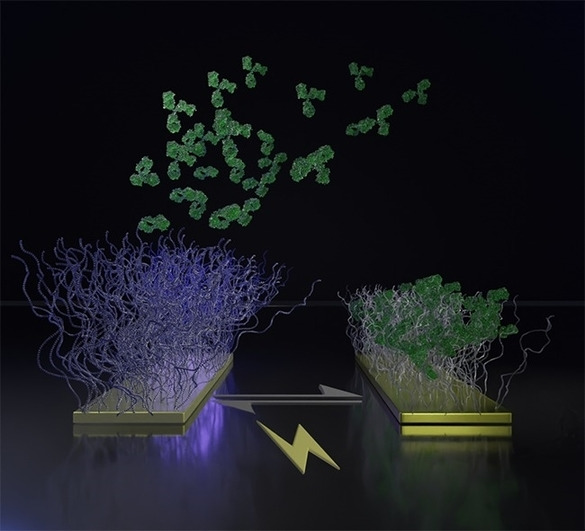

## Introduction

Biointerfaces are the boundaries between living and artificial environments. The chemical design of biointerfaces is essential as it provides functioning implants, cell cultures, neural bioelectronics and various analytical devices in direct contact with a biological environment. In this context, synthetic polymers attached to surfaces have proven suitable for a wide range of applications, such as biomolecule purification, protein arrays, antibacterial coatings and drug‐delivery.^[1]^ In particular, so called polymer brushes formed by end‐grafted chains at high surface density^[1f]^ are interesting because they can immobilize large amounts of proteins by non‐covalent reversible interactions^[2]^ while also preserving their structure and biological activity.^[3]^ Conversely, an equally important function of polymer brushes is to act as barriers that prevent biomolecular adsorption and cell attachment,[^1b^, ^1f^] so called antifouling surfaces. In some cases, a polymer brush may switch between an attracting and a repelling state with respect to a certain biomolecule, depending on factors in the liquid environment such as temperature,^[4]^ salt content^[5]^ or pH.^[5]^ In principle, this is highly interesting for applications as it creates responsive interfaces[^1a^, ^1d^, ^1f^] that may be used to capture and subsequently release proteins for instance.

Despite these appealing features, however, applications of responsive polymer brushes remain limited because bulk liquid properties cannot (or should not) be changed in living systems. The surface of a device used for implantation or wound dressing will be continuously exposed to the same physiological environment,^[6]^ which means that even if its interface has some form of responsive behavior, it cannot be utilized. This has led to the development of various electrochemical methods that attempt to *locally* manipulate the chemically modified interface.^[7]^ Electrochemical control is appealing since it requires little power, is compatible with miniaturized systems^[8]^ and offers remote control of implanted devices.^[9]^ Still, to date it has proven very difficult to electrically switch an interface between, for instance, a binding and a repelling state with respect to proteins, especially in a reversible manner. Several chemical constructs have been specifically developed for the immobilization and on‐demand electrochemical release of specific small molecules,^[10]^ DNA^[11]^ and whole cells.^[12]^ Yet, in terms of proteins, only a few examples exist showing release of monolayers of electrostatically adsorbed insulin^[13]^ or His‐tagged protein A.^[14]^ To begin with, none of these concepts can be compared with protein immobilization in polymer brushes in terms of binding capacity and structure preservation. Furthermore, the chemistry is irreversibly altered upon electrochemical release, which means that devices are limited to single use. Most importantly, no device has been shown to work in an actual biological environment. For instance, it remains impossible to controllably release a protein (e.g. a therapeutic antibody) directly into a bodily fluid by electrochemical control. This is currently a major drawback for future biomedical technologies since protein biologics now constitute most new therapeutic drugs, while being particularly challenging to produce and administer.^[15]^

In this work we present the first generic protein capture–release system that can be electrochemically controlled and functions under biological conditions. Our interface design provides high protein binding capacity, while still being able to release any desired amount of the immobilized molecules on‐demand. The degree of repulsion can be tuned electrically, showing selective protein uptake or zero adsorption while in contact with biofluids. Furthermore, we show that a glucose‐mediated reduction in pH can be maintained at the interface, at fully physiological conditions, even in the presence of buffering species in the bulk. We present several applications of our technology, with particular emphasis on controlled release of biologics with tunable doses inside a biological environment, a particularly critical milestone for future biomedical devices.

## Results and Discussion

**Figure 1:**
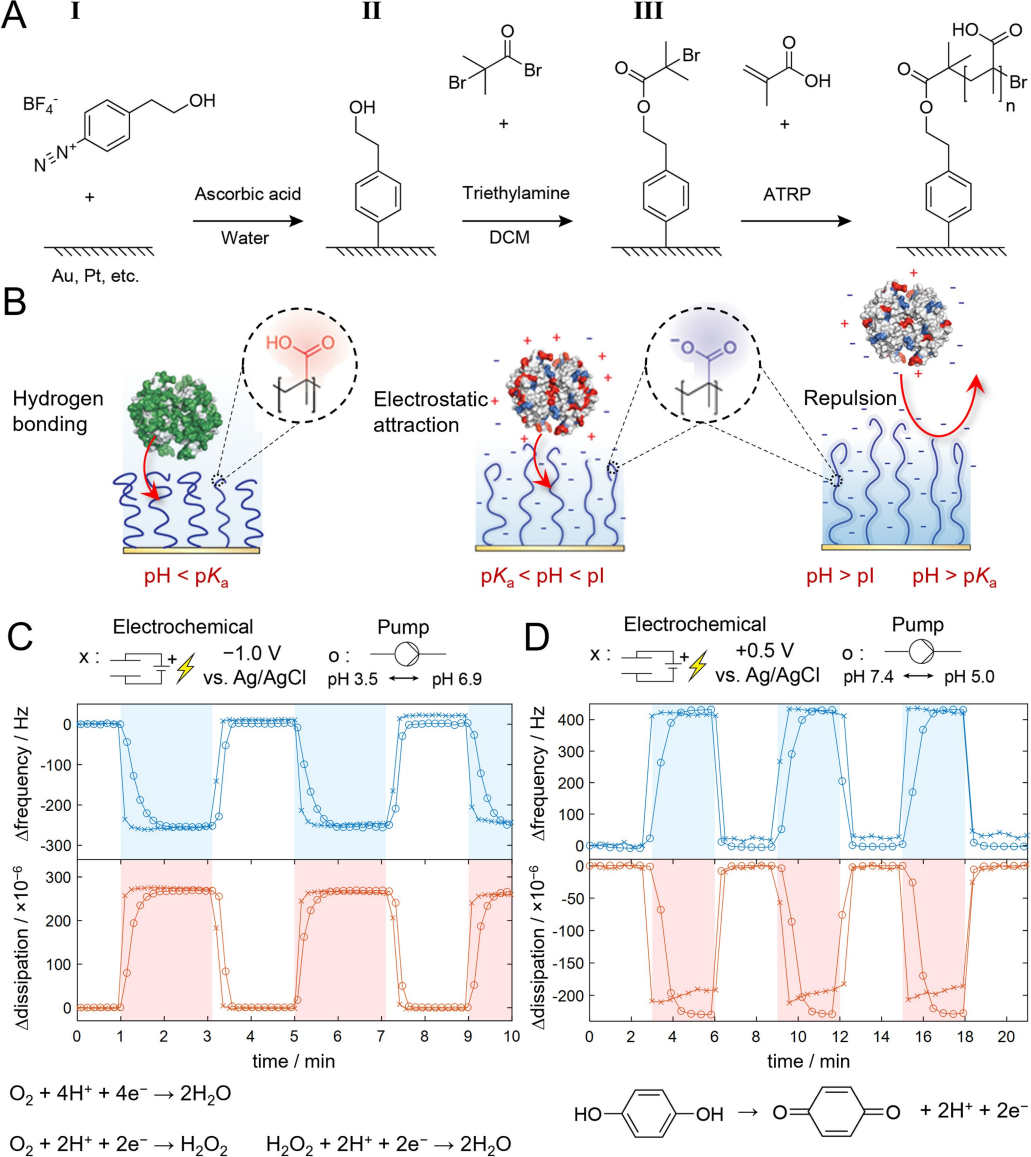
Interface preparation and electrochemical switching. A) Synthesis scheme for polymer brushes grafted via aryl bonds using reduction of a diazonium salt. B) States of the brush: generic hydrogen bonding state (pH≤5 at physiological ionic strength), electrostatically attracting state (if the protein has high pI) and fully repelling state (when pH is sufficiently high). C) Electrochemical QCMD data of brush switching in a pH 5.0 buffer based on proton consumption by reduction of ambient O_2_. The voltage is switched on in the indicated intervals. The response is the same (but slower) when changing the bulk pH by flowing different buffers over the surface. D) Electrochemical brush switching in the opposite direction at physiological pH. Acidification occurs by oxidation of hydroquinone (5 mM). Again, the response from altering pH by buffer exchange is shown for comparison.

The design of the electrochemically controllable polymer brush interface is outlined in Figure 1. In the first and most crucial step, a diazonium salt^[16]^ is synthesized (Figure S1) and reduced by ascorbic acid to generate a covalent link to the electrode, forming a very thin layer that leaves the metal accessible. Next, this film is converted to an initiator layer by bromoisobutyrate, which enables activator‐regenerated atom transfer radical polymerization^[1e]^ (ATRP) to be performed as described previously^[17]^ (Figure 1A). A very large variety of polymers can be synthesized by ATRP,^[1e]^ but throughout this paper we only present results on poly(methacrylic acid) (PMAA) brushes. We used a polymerization time that resulted in PMAA brushes with thickness in the range of tens of nm in the dry state and a few hundred nm when hydrated, as determined by surface plasmon resonance^[17]^ (SPR). The degree of hydration is ≈80 % in the neutral state and ≈90 % in the ionized state.^[3b]^ Figure 1B shows the three pH‐dependent states for how proteins can interact with or be repelled by the polyacidic brush. First, in their neutral state, PMAA brushes are known to efficiently bind water‐soluble proteins in a structure‐preserving manner, an effect we have attributed to hydrogen bonds.^[3b]^ Note that it is sufficient to go down to pH 5 to protonate the brush almost fully (≈10 % ionized groups).^[18]^ At physiological or higher pH (and bodily salt content), the brushes are charged and may bind proteins by electrostatic attraction if the isoelectric point (pI) is sufficiently high.[^3b^, ^19^] Finally, once the pH is high enough to make both protein and polymer negatively charged, the brushes become protein repelling.

Remarkably, we found that our surface functionalization protocol provided a polymer anchor which was stable during electrochemical potential sweeps, while Faradaic reactions still could occur efficiently. Electrochemical quartz crystal microbalance with dissipation monitoring (QCMD) was used as a sensitive tool to probe the degree of hydration of the brush as it changed ionization state.^[18]^ Both the frequency and the dissipation response were the same when a potential was applied as when performing liquid exchange with buffers that had pH well above and below the brush p*K*_a_ ^[18]^ (around 6.2 at physiological salt). Notably, the electrochemical switching was very fast (≈10 s), even in the buffered environment, and fully reversible (tested up to ≈100 cycles). The brushes could be pH‐switched in both directions around their p*K*_a_: either by a negative potential in an environment buffered at pH 5 (Figure 1C) or by a positive potential in a physiological buffer at pH 7.4 (Figure 1D). The reductive switching relies simply on ambient oxygen,^[20]^ which was confirmed by a weaker responsive behavior in buffers purged with nitrogen (Figure S2). The oxidative switching was achieved by addition of species that produce protons upon oxidation, such as hydroquinone^[21]^ or others (Figure S3). We observed no major influence from the liquid flow rate in the cell on the switching capability. This is important as it shows that the responsive nature of the interface remains even when there is convection in the surrounding liquid.

The fast and complete switching behavior is in good agreement with theory (details in Supporting Information). In brief, the local pH is a function of time and distance from the surface and can be modelled by partial differential equations that describe the mass transport (in the high potential limits). Our models showed that the pH quickly (within seconds) reaches quite extreme values inside the thin region occupied by the brush (Figure S4). For instance, with a bulk pH of 7.4, we predicted that reductive potentials can increase the local pH to ≈12, i.e. higher than the pI of any protein. Furthermore, we estimated that in the presence of a few mM redox‐active species, an oxidative potential can reduce the pH to ≈3, which is more than enough to fully protonate the PMAA brush.^[18]^

We emphasize that the key to succeeding with an electrochemically responsive interface design is not only the polyelectrolyte brush, but also the grafting chemistry. Small molecules can diffuse through the hydrated brush and undergo Faradaic reactions efficiently at the electrode, while there is no damage or detachment of the grafted chains. Both these features are critical and together they enable us to present complete and reversible electrochemical brush switching for the first time. Indeed, the switching behavior could not be achieved with other strategies concerning the chemistry. For instance, we found that electrografting, which is the established procedure for attaching aryl groups by reducing diazonium,^[22]^ produced multilayers that later prevented Faradaic reactions (Figure S5). Furthermore, thiol‐based anchoring resulted in rapid polymer desorption at negative potentials (Figure S6), in agreement with previous observations.^[23]^ Furthermore, the aryl‐based grafting can be performed on a great variety of electrode materials^[24]^ (gold and platinum in this work). Next, we will present applications enabled by our electrochemically responsive brush interface, starting with zero fouling on demand in a biological environment.

**Figure 2:**
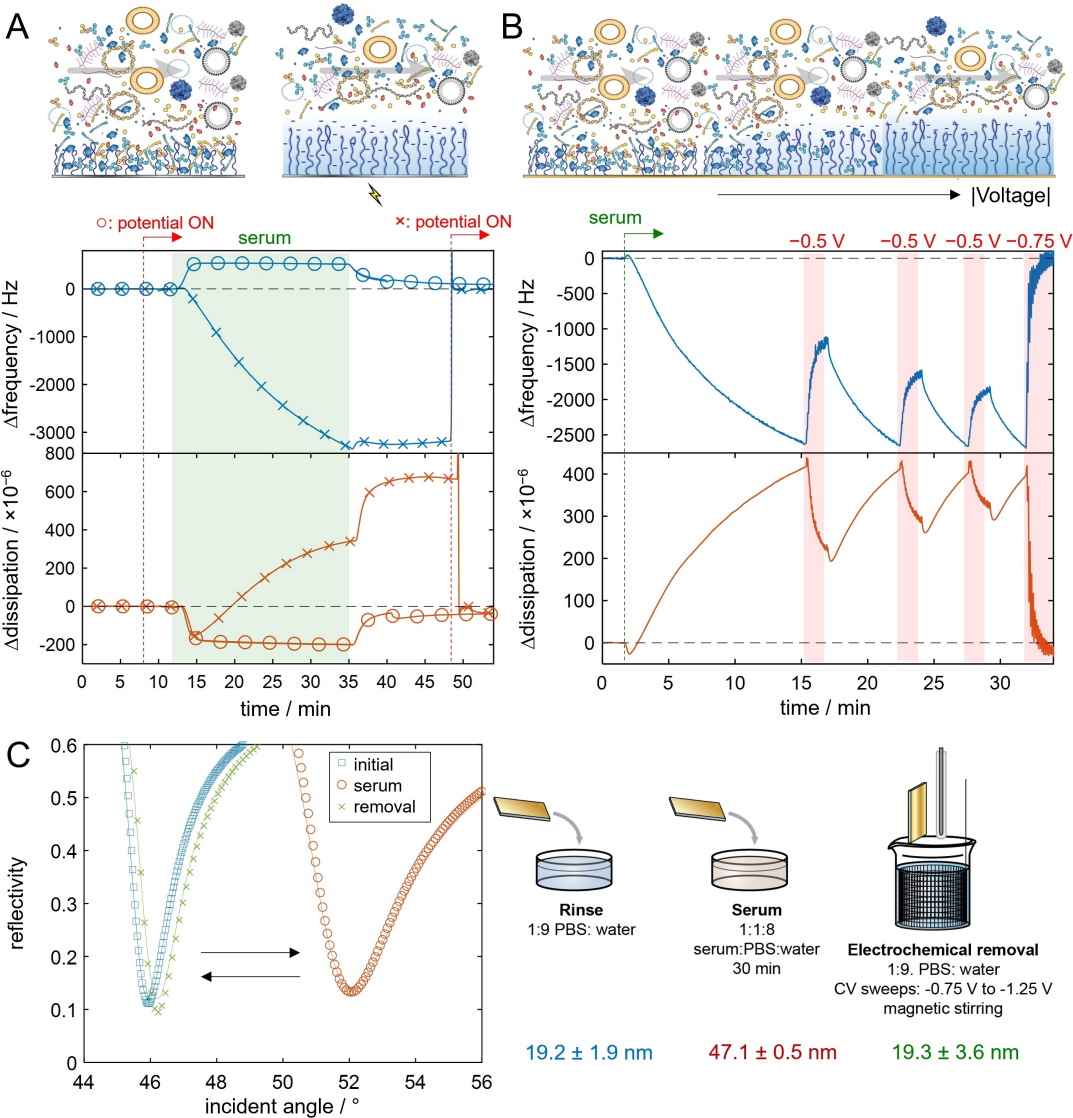
Zero fouling on demand and selective protein binding. A) QCMD data of brushes (on Pt) exposed to serum in PBS pH 7.4 (from 12 to 35 min). Crosses: Potential applied after serum proteins have bound ‐ the baseline is recovered. Circles: Potential applied before serum is introduced ‐ no binding is detected (the small step‐like response is due to the bulk viscosity change). Potential “on” means repeated sweeps from 0 to −0.5 V at 200 mV s^−1^. B) Electrochemical tuning of adsorbed protein amount (brushes on Au). Note that the surface remains in contact with serum. More proteins are released as a stronger reductive potential is used: 25 sweeps to −0.5 V at 200 mV s^−1^ leads to partial release (3 repeats) and 75 sweeps to −0.75 V at 200 mV s^−1^ leads to complete release. C) Confirmation of full electrochemical release of serum proteins by SPR spectra in dry state (Au surfaces). The thicknesses are those determined using Fresnel models (solid lines), assuming a refractive index of 1.5 for the organic coating (polymer and protein). Any remaining protein amount is within the measurement uncertainty when remeasuring.

High requirements are placed on electrodes to remain functional in biological environments for applications such as sensing^[25]^ or neuron stimulation.^[26]^ We exposed our brushes to complete serum and evaluated their non‐fouling properties (Figure 2). The serum was diluted 10× simply to enable flow, but the solutions still had a very high total protein concentration (pure plasma has 100 g L^−1^ ^[27]^). In most biological environments, the brush will obtain a negative charge since pH > p*K*_a_ and ternary adsorption may occur, in particular through electrostatic interactions for proteins that are positively charged.^[19]^ Indeed, upon exposure to serum at pH 7.4 with the potential off, massive amounts of biomolecules bound to the brushes (thousands of Hz), yet the baseline was fully recovered when a cathodic potential was subsequently applied (Figure 2A). Importantly, we could also perform an electrochemical “cleanup” of the interface while it was still inside the serum environment (Figure 2B). As stronger and stronger reductive potentials were applied, more proteins were removed and eventually the baseline was again recovered, even in the presence of serum. We emphasize that this switching to a non‐fouling state relies entirely on ambient O_2_, i.e. no redox‐active species were added. To further confirm that all serum proteins could be electrochemically removed from the brushes, we measured SPR spectra in air^[3b]^ after binding and release (Figure 2C). The protein removal efficiency was the same when comparing the electrochemical cleaning with dipping of surfaces in a pH 11.5 buffer (Figure S7). Clearly, the high quantity of bound proteins (≈4 μg cm^−2^ by SPR) does not hinder transport of naturally present O_2_ from the biofluid to the electrode.

The strong protein repulsion when the interfacial pH is increased is partly because hydrophilic brushes (neutrally charged) normally repel proteins due to entropic effects from hydration and chain conformation.^[1b]^ Here the electrochemical control is used to introduce additional repulsion by electrostatic forces, thereby apparently overcoming any attraction entirely. The possibility to fully prevent fouling simply by occasionally applying low potential pulses enables new possibilities for interfaces in biotechnology, for instance on implanted devices. The energy requirements are very low (9 μW cm^−2^ during the potential sweeps), showing compatibility with small power sources.

As shown by the data in Figure 2B, more proteins are removed when the reductive potential is stronger and applied for a longer time. This means that the voltage determines which proteins can be electrostatically attracted to the brush through tuning of the interfacial pH. Indeed, the voltage required for releasing a specific protein also depended on the buffering capacity of the solution (Figure S8), but full release was possible in standard PBS buffer for all proteins tested (Table S1). Another trend that could be observed was that proteins with higher pI tended to bind more strongly to the brush, as expected,^[2a]^ although other factors such as molecular weight also influenced the affinity. Regardless, the electrochemical control clearly provides means to select which proteins bind to the brush in a much more direct manner compared to changes in the bulk liquid environment. This can be useful for separation processes or protein purification, for instance in the production of biologics.^[28]^ At the same time, zero fouling can be achieved at any time by applying the strongest reductive potential (around −1.0 V vs. Ag/AgCl depending on electrode type) and a generic protein binding state^[3b]^ can be induced by lowering the pH to 5 (Figure S9), although the latter requires the presence of proton producing redox‐active molecules.

**Figure 3:**
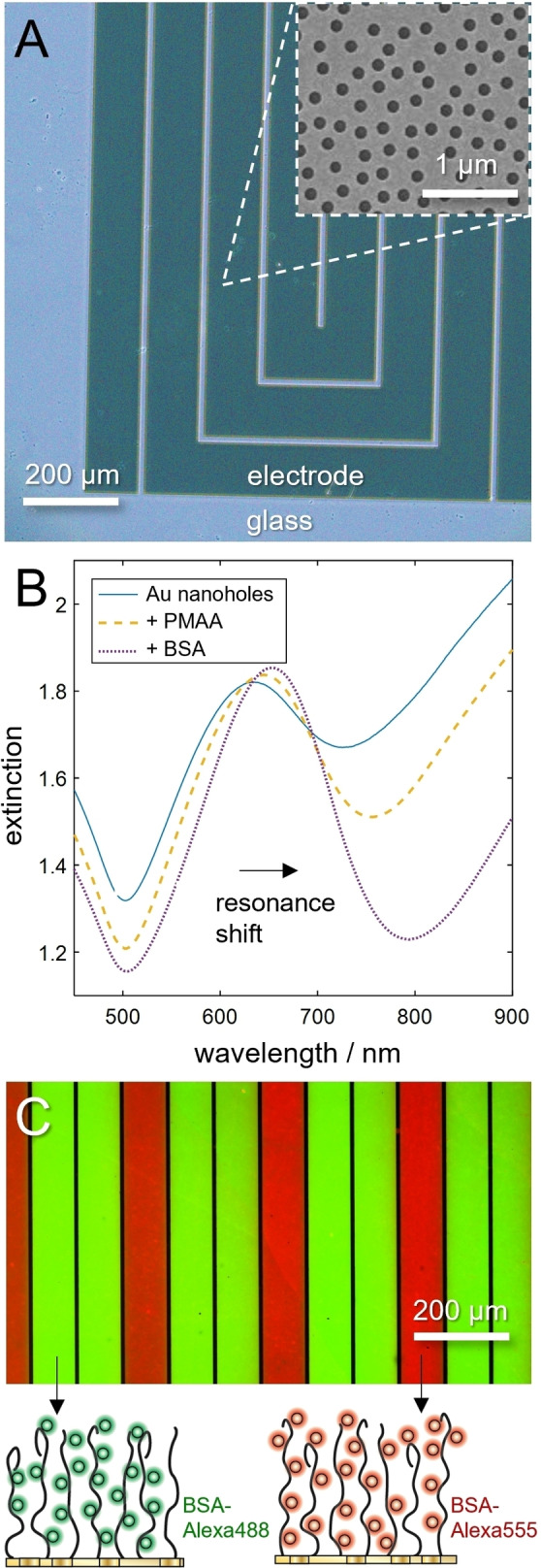
Protein patterning on microelectrodes. A) Microscope photo of electrodes and scanning electron microscopy image of the plasmonic nanoholes in 30 nm Au. B) The resonance in the extinction spectrum in air confirms brush synthesis and protein immobilization on the nanostructured surface. C) Superimposed fluorescence images measured from the microelectrode stripes after localized release of green‐labelled BSA and a second immobilization step of red‐labelled BSA.

Since the electrochemically induced pH changes are localized to the surface, they can also be utilized to selectively bind proteins to micropatterned electrodes, i.e. the control is both in space and time. As an example of pattern generation, we used photolithography to prepare gold electrodes in 100 μm stripes, which also contained plasmonic nanoholes (Figure 3A for label‐free optical detection of binding in transmission mode (Figure 3B).^[29]^ Fluorescence was used to verify the patterning strategy by first introducing bovine serum albumin (BSA) labelled with green fluorophores to the whole surface, followed by desorption from selected electrodes and refilling with BSA labelled in red (Figure 3C). This patterning method can be used for creating protein biochips or other functional arrays.^[30]^ Besides the high‐capacity and non‐invasive immobilization by the brush,^[3b]^ it provides an alternative to microdispensing, thereby avoiding the well‐known issues associated with drying of small droplets.

**Figure 4:**
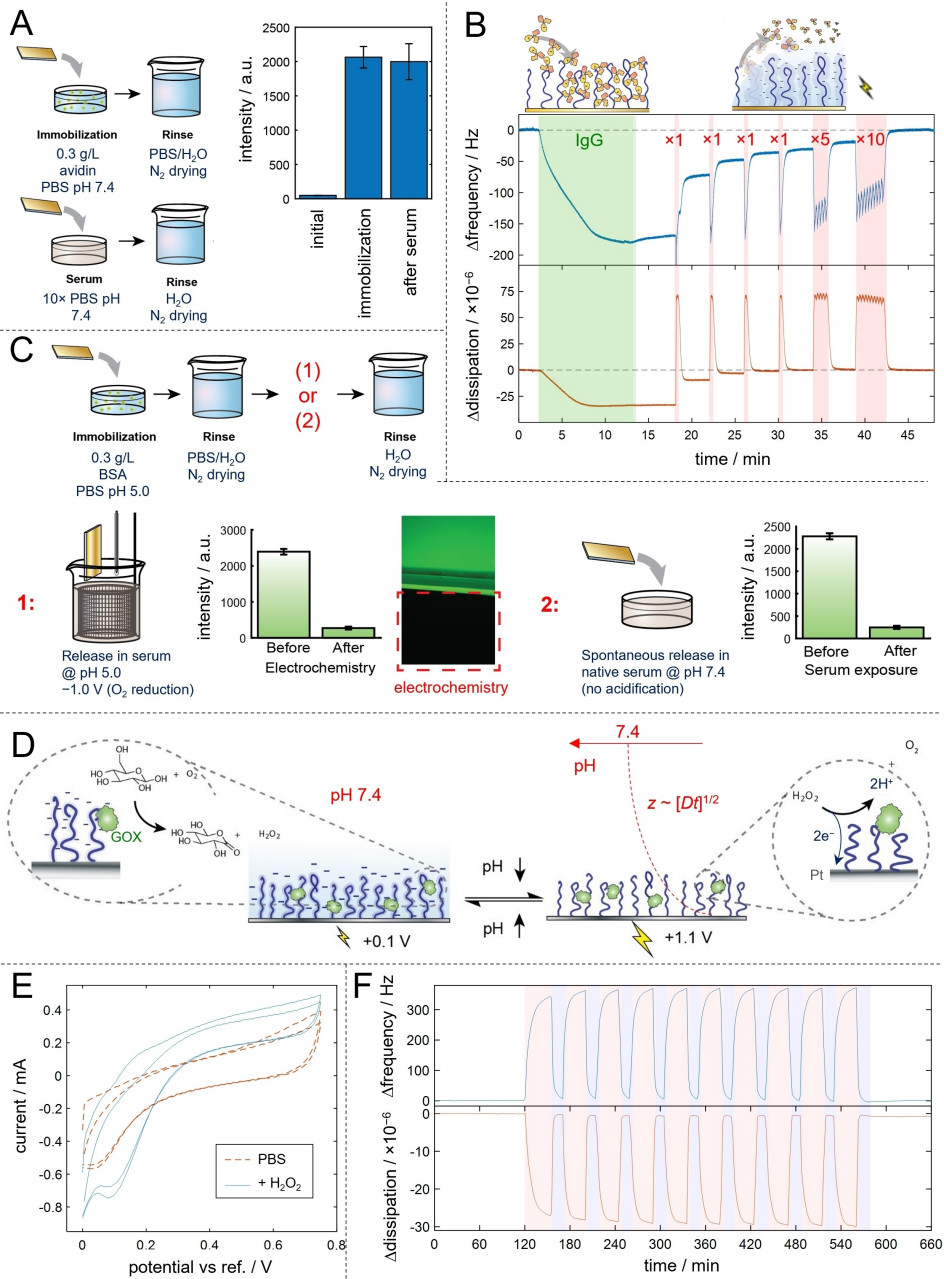
Electrically controlled and tunable release of proteins in biological settings. A) Supporting data for the delivery strategy based on electrostatic interactions at pH 7.4 and release by native O_2_ (see also Figure 2). The fluorescent intensity from electrostatically immobilized labelled avidin is measured before and after exposure to serum (30 min) at fully physiological conditions. B) QCMD data showing immobilization and tunable release of an IgG antibody (bulk pH 5.0). Cathodic potentials are applied by sweeps from 0 to −0.5 V with the number of sweeps indicated at each release event. C) Delivery strategies utilizing hydrogen bonds, tested with BSA. First, electrochemical release can be performed in serum with pH lowered to 5 as verified by fluorescence imaging. This strategy requires a locally lowered pH in the biological system. Second, spontaneous release occurs in serum at pH 7.4. This strategy requires an ongoing acidification (which is switched off for release). D) Principle of local acidification on Pt electrodes with PMAA brushes, powered by glucose and mediated by covalently bound enzymes. The interfacial pH is kept low by electrochemical oxidation of H_2_O_2_ produced from the glucose breakdown (no redox active species added). E) Redox activity of H_2_O_2_ (5 mM) during anodic sweeps on Pt surfaces functionalized with PMAA brushes. The current in the absence of H_2_O_2_ is shown for comparison. F) Experimental verification of brush switching based on the concept in panel D using 10 mM glucose (no redox active species introduced). The potential is always on but switches between +0.1 and +1.1 V.

Next, we show how electrically responsive polymer brushes can be used for controlled release of immobilized proteins in a biological setting, i.e. electrochemical delivery of biologics. First, it is clear from previous results (e.g. Figure 2) that at pH 7.4, i.e. the pH in most regions of the human body, electrostatic interactions can be used to immobilize certain proteins and release them simply by utilizing ambient O_2_. To confirm this strategy for controlled release, we verified that desorption did not occur when brushes with positively charged proteins were exposed to serum proteins at fully physiological conditions (Figure 4A). However, most proteins do not have a very high pI (antibodies are in the range 6–8) and bound poorly or not at all to the brushes at pH 7.4. If one still wants to utilize electrostatic interactions, the protein of interest will likely need to be modified with a polycationic tag. For instance, poly(L‐lysine), which is established for drug delivery applications,^[31]^ can be conjugated to the biologic^[32]^ or in some cases perhaps even engineered into its sequence. Still, the hydrogen bonds at pH≤5 are generic, structure‐preserving and stronger than electrostatic interactions at physiological salt,^[3b]^ making them preferable for a delivery strategy that should work for any water‐soluble protein. Therefore, we also developed methods for controlled release of proteins immobilized by hydrogen bonds.

As a first step, we confirmed that high‐capacity immobilization and release in controlled doses of an IgG antibody, the most common biologics, was possible (Figure 4B). Next, we verified that electrochemical release based on O_2_ reduction was possible also in a serum environment if the pH was adjusted to 5 (Figure 4C). Also, as the immobilized proteins were exposed to serum at pH 7.4, release occurred spontaneously, as expected since the hydrogen bonds are broken when the polymers become ionized.^[3b]^ Importantly, these results show that other biomolecules in the environment do not interfere with the release process. Based on this finding, one can identify two different strategies to release proteins immobilized by hydrogen bonds. First, if a pH of about 5 is maintained in a passive manner at the interface in contact with the biofluid, the proteins can be released when a reductive potential is applied. Such low local pH may be maintained in vivo by spontaneous degradation of biocompatible polymers.^[33]^ The alternative strategy is to use electrical control for local acidification inside a biological environment. In such a “reversed” delivery strategy, electrochemistry is used to keep the pH lowered at the interface, so that release occurs by spontaneous breaking of hydrogen bonds when the system is left idle (Figure 4C). The issue with this approach is that the biofluid may not contain any suitable proton producing redox‐active species at sufficient concentrations.

To address this remaining challenge, we present an acidification method based on enzymatic glucose breakdown (Figure 4D). The key idea is that glucose is spontaneously broken down by glucose oxidase (GOX) into D‐glucono‐1,5‐lactone and H_2_O_2_.^[19]^ To generate protons, we utilize the spontaneous breakdown of H_2_O_2_ on platinum electrodes and subsequent acidification by electrochemical oxidation of hydrated Pt surface sites.^[20a]^ SPR measurements on 20 nm Pt films^[34]^ were used to verify similar ATRP growth rates as on Au (Figure S10). We found that, analogously to hydroquinone for Au, a H_2_O_2_ concentration of a few mM caused clear Faradaic reactions (Figure 4E) and was sufficient to observe switching of the PMAA brushes (see also Figure S11). A relatively small amount of GOX was covalently bound to PMAA,^[19]^ leaving most of the ‐COOH groups in the brush unmodified and available for immobilization of other proteins, as evident from the remaining pH‐responsive behavior after GOX conjugation (Figure 4F). Supplementary experiments showed similar switching when GOX was introduced in solution instead (Figure S12). It should be noted that in these proof‐of‐concept experiments, the glucose concentration was 10 mM, which is higher than but still comparable to normal values in blood (less than a factor of two). The exact required glucose concentration will in the end depend on many factors, in particular electrode geometry, which was limited to planar surfaces in this initial study. In summary, the GOX results show yet another strategy for controlled release of proteins from the PMAA brushes, based on enzymatic catalysis and “powered” by glucose from the biological environment.

## Conclusion

We have presented the first method for electrochemically controlled reversible catch and release of proteins via pH changes at a polymer brush interface. Our concept gives perfect contrast, i.e. no adsorption is detected in the repelling state, and enables excellent control of protein binding in space and time. Using hydrogen bonds, the method is applicable to all water‐soluble proteins, which immobilize in large amounts in a manner that preserves their structure.^[3b]^ We show several application areas, such as zero fouling on‐demand, pattern formation and controlled release of proteins in a biological environment. The latter can be particularly useful for novel devices that aim to stimulate the human body, such as smart wound dressings. Future work will also upscale the technology into 3D by preparing polymer brushes on porous materials,^[35]^ which would enable implementation in large‐scale separation technologies and other analytical devices. We point out that this pioneering study has utilized the single polymer PMAA and its interactions with proteins (hydrogen bonds or electrostatic). Considering the great variety of polymers that can be synthesized by ATRP in the same manner^[1e]^ and the broad range of supramolecular interactions with biomolecules, this opens up for many future studies on electrochemically controlled capture and release of biomolecules (not limited to proteins). For instance, affinity‐based capture with high selectivity is possible by covalently immobilizing receptors in the polymer brush, similarly to how GOX is bound in this work or by other means. This can be followed by controlled electrochemical release as long as the affinity has a pH dependence. Indeed, raising the pH is a standard method for breaking non‐covalent biomolecular interactions, which speaks for the broad applicability of this technology.

## Conflict of interest

The authors G.F.D.dC. and A.D. have filed a patent based on the findings presented here and started the company Nyctea Technologies AB.

1

## Supporting information

As a service to our authors and readers, this journal provides supporting information supplied by the authors. Such materials are peer reviewed and may be re‐organized for online delivery, but are not copy‐edited or typeset. Technical support issues arising from supporting information (other than missing files) should be addressed to the authors.

Supporting Information

## Data Availability

The data that support the findings of this study are available from the corresponding author upon reasonable request.
