# Supplementary material for: Electrically Switchable Polymer Brushes for Protein Capture and Release in Biological Environments
Source: Angew Chem Int Ed Engl. 2022 Mar 30;61(22):e202115745. doi: 10.1002/anie.202115745 (PMC9311814; doi:10.1002/anie.202115745)
Supplement: Supplementary file 1 — Supporting Information [file ANIE-61-0-s001.pdf]

Angewandte Chemie
Eine Zeitschrift der Gesellschaft Deutscher Chemiker

Supporting Information

Electrically Switchable Polymer Brushes for Protein Capture and Release in Biological Environments

*G. F.-D. del Castillo, M. Kyriakidou, Z. Adali, K. Xiong, R. L. N. Hailes, A. Dahlin**

Supporting Information
©Wiley-VCH 2022
69451 Weinheim, Germany

Electrically Switchable Polymer Brushes for Protein Capture and Release in Biological Environments

Gustav Ferrand-Drake del Castillo, Maria Kyriakidou, Zeynep Adali, Kunli Xiong, Rebekah L. N. Hailes and Andreas Dahlin*

Abstract: Interfaces functionalized with polymers are known for providing excellent resistance towards biomolecular adsorption and for their ability to bind high amounts of protein while preserving their structure. However, making an interface that switches between these two states has proven challenging and concepts to date rely on changes in the physiochemical environment, which is static in biological systems. Here we present the first interface that can be electrically switched between a high-capacity (>1 µg/cm^2) multilayer protein binding state and a completely non-fouling state (no detectable adsorption). Switching is possible over multiple cycles without any regeneration. Importantly, switching works even when the interface is in direct contact with biological fluids and a buffered environment. The technology offers many applications such as zero fouling on demand, patterning or separation of proteins as well as controlled release of biologics in a physiological environment, showing high potential for future drug delivery *in vivo*.

DOI: 10.1002/anie.202115745

Table of Contents

Experimental section	Page 3
Figure S1	Page 5
Figure S2	Page 5
Figure S3	Page 6
Figure S4	Page 6
Figure S5	Page 7
Figure S6	Page 8
Figure S7	Page 9
Figure S8	Page 10
Figure S9	Page 11
Figure S10	Page 11
Figure S11	Page 12
Figure S12	Page 13
Table S1	Page 14
Theory	Page 15
References	Page 16

Experimental Section

Chemicals: All chemicals and proteins used were purchased from Sigma-Aldrich unless stated otherwise. Water was ASTM research grade Type 1 ultrafiltered water (milli-Q-water). H_2O_2 (30%) and NH_4OH (28-30%) were from ACROS, while H_2SO_4 (98%) and ethanol (99.5%) were from SOLVECO. Chemicals used for brush synthesis were 4-aminophenethyl alcohol, tetrafluoroboric acid (48% solution in water), acetonitrile, tert-butyl nitrate, diethyl ether, L-ascorbic acid, dichloromethane, triethylamine, α-bromoisobutyryl bromide, tert-butyl methacrylate, dimethylsulfoxide, dichloromethane, methane sulfonic acid, N,N,N',N''-pentamethyldiethylenetriamine (PMDTA) and $CuBr_2$. Buffers used in this work were based on phosphate buffered saline tablets (0.01 M phosphate, 0.13 M NaCl, pH 7.4) or disodium hydrogen phosphate and NaCl titrated to a specific pH with HCl (1 M aqueous solution) or NaOH (1 M aqueous solution). The proteins used in this study were avidin (ThermoFisher), avidin-fluorescein isothiocyanate conjugate (ThermoFisher), bovine serum albumin (BSA), BSA-fluorescein isothiocyanate conjugate, fibrinogen from bovine plasma, glucose oxidase Type VII G2133 from Aspergillus Niger, horse radish peroxidase (ThermoFisher), insulin, insulin glargine, IgG antibodies from human serum, lysozyme, NeutrAvidin (ThermoFisher), ribonuclease A, proteinase K, α-chymotrypsin and lactoferrin. Alexa FluorTM 488 and 555 labeling kits were from ThermoFisher. Human serum (from human male AB plasma) was filtered through a 40 µm hydrophilic filter and diluted ten times prior to use. Fouling tests were performed with serum diluted in either water or PBS, while experiments on controlled release were always performed with serum diluted in PBS at full buffer strength. The redox active species tested were hydroquinone, dopamine hydrochloride (DOPA), ascorbic acid, 4-aminophenethyl alcohol (tyrosol), 3,4-dihydroxyphenetylacetic acid (DOPAC), and β-nicotinamide adenine dinucleotide, reduced disodium salt hydrate (NADH).

Diazonium salt synthesis: The synthesis of diazonium salt (Figure S1) involved a modified literature procedure.[1] Under an inert atmosphere, 4-aminophenethyl alcohol (2.94 g, 20 mmol) and tetrafluoroboric acid (48% solution in water, 9.94 g, 113 mmol) were dissolved in acetonitrile (20 mL). In a separate flask, tert-butyl nitrate (2.269 g, 22 mmol) was dissolved in acetonitrile (12 mL). Both solutions were degassed and cooled to −20 ºC alongside 200 mL of diethyl ether. After 20 min the solutions were warmed to 0 ºC, before the tert-butyl nitrate solution was added to the 4-aminophenethyl alcohol solution dropwise with stirring. The reaction was then stirred for a further 1 h. The reaction was terminated by dropwise addition of the dark yellow solution to rapidly stirring diethyl ether (200 mL). After additional stirring for 1 h the supernatant was decanted off. The brown colored precipitate was dried and 3.69 g of impure diazonium salt was obtained and carried forward without further purification. To verify the product, 1H NMR spectra were recorded at ambient temperature on a Varian 400 MHz NMR spectrometer. Spectra were analyzed relative to external tetramethylsilane and were referenced to the most downfield residual solvent resonance (CDCl3: δH 7.26 ppm). 1H NMR resonances of the diazonium salt matched those previously reported[1] and analysis revealed a purity of 80%.

Surface cleaning: Prior to surface functionalization, QCM sensor crystals (standard Au, purchased from Biolin Scientific) and SPR sensor surfaces (standard Au, purchased from Bionavis) were cleaned with piranha wash (H_2SO_4:H_2O_2, 3:1 v/v) for 10 min followed by rinsing in milli-Q. Next, an RCA1 wash (H_2O:H_2O_2:NH_4OH 5:1:1 v/v at 75 °C) was performed for 20 min, followed by further rinsing in milli-Q, sonication in ethanol, and drying with N_2. For microelectrodes the piranha wash step was omitted to prevent destruction of the surface due to delamination of the gold film with nanoholes.

Surface activation: Gold or platinum surfaces (QCM and SPR sensors) were placed in a glass jar with a septum seal containing diazonium salt 1 (0.301 g, 1.28 mmol) and the jar was purged with N_2. In a separate flask, ascorbic acid (0.028 g, 0.16 mmol) was dissolved in water (40 mL) and the solution was degassed for 1 h. Then, the ascorbic acid solution was transferred into the sealed glass jar causing reduction of the diazonium salt. The gold surfaces were stirred in the solution for 1 h by use of a platform shaker (nitrogen bubbles that appear on the surface after 15 min indicate successful diazonium salt monolayer formation), after which they were thoroughly rinsed in water then ethanol and dried. To convert the diazonium monolayer into a polymerization initiator layer, the gold surfaces were exposed to α-bromoisobutyryl bromide (0.222 mL, 1.80 mmol) and triethylamine (0.302 mL, 2.17 mmol) in dichloromethane (20 mL) for 10 min, after which surfaces were rinsed in ethanol and dried under N_2.

Surface-initiated polymerization: ATRP was used to prepare PMAA polymer brushes similarly to established protocols. Inhibitor was removed from the monomer tert-butyl methacrylate using an alumina column, after which it was stored at −20 °C, then warmed to room temperature immediately before use. Reactions were carried out using standard Schlenk line techniques under an inert atmosphere of N_2. $CuBr_2$ (0.006 g, 0.03 mmol), and PMDTA (0.052 mL, 0.246 mmol) were dissolved in dimethyl sulfoxide (20 mL) and, alongside a separate flask of tert-butyl methacrylate (20 mL, 0.1231 mol), was deoxygenated via vigorous bubbling of N_2 for 30 min. The reaction solution and monomer were then transferred via cannula into a screw-top jar (with rubber septa lid) containing initiator-prepared gold surfaces. The reaction was initiated by the addition of ascorbic acid (0.033 g, 0.185 mmol). The final concentrations of each component in the reaction medium were: [monomer] = 3.1 M, [$CuBr_2$] = 0.6 mM, [PMDTA] = 6.2 mM, and [ascorbic acid] = 4.6 mM. The reaction was placed under magnetic stirring. Reactions were quenched by immersing the samples in pure ethanol. Poly(tert-butyl methacrylate) brushes were then converted to PMAA by exposure to 0.2 mM methane sulfonic acid in dichloromethane (10 mL) for 15 min, followed by rinsing in dichloromethane and ethanol.

Quartz crystal microbalance: Sensor crystals coated with gold were used and measurements were performed using a Q-Sense E4 (Biolin Scientific). All frequency and dissipation data shown corresponds to the fundamental resonance at ~5 MHz (no overtones). A flow cell with an electrochemical module (QEM 401) was used to perform in-situ electrochemical experiments. A Gamry Interface 1000E potentiostat (Gamry Instruments) was connected to the electrochemical cell. For every experiment the internal resistance of the circuit was measured (Get Ru) and the open circuit potential was measured to verify an acceptable reference electrode state and correctly connected circuit. The Ag/AgCl reference electrode used was a World Precision Instrument low leakage "Dri-ref" electrode. Potentials were applied either by chronoamperometry (fixed voltage) or by voltammetry sweeps with a rate of 100 mV/s unless otherwise specified. The active electrode area is 0.78 cm^2 for all current data shown. All voltages stated are vs the reference electrode.

Surface plasmon resonance: Measurements were performed on a SPR Navi 220A instrument (BioNavis), both in air and in water. The total internal reflection (TIR) and SPR angle was recorded on three different laser wavelengths and in two different flow channels. The flow rate of buffer used was 20 µL/min. Electrochemical SPR measurements were performed by connecting a potentiostat (same as for QCMD) to a cell designed for this purpose (from the instrument manufacturer). The methodology of analyzing SPR spectra by Fresnel modelling and the quantification has been described in previous work.[2] In brief, the refractive index of the dry polymer brushes was set to 1.522. The deposited diazonium layer was assumed to have refractive index 1.5 (typical for organic coatings). The refractive index of the proteins was assumed to be equal to that of the polymer. To obtain surface coverage, the densities of the dry polymers and proteins were used (1.22 g/cm^3 and 1.35 g/cm^3 respectively).

Plasmonic detection with nanohole arrays: Extinction spectroscopy was performed to detect the shift of the resonance peak from nanohole arrays in 30 nm gold films.[3] In brief, the surface was illuminated by a tungsten lamp and a fiber coupled photodiode array spectrometer (B&WTek) analyzed the spectrum. (A small fraction of the light was directly transmitted through the glass in between the electrodes, but this only influences the absolute extinction values, not the resonance shift.) Note that for plasmonic nanohole arrays the signal is a spectral shift in nm, while SPR uses angular shifts in degrees.

Fabrication of microelectrodes: To create microscale stripe electrodes, a laser writer (Heidelberg Instruments DWL 2000) was used. The photoresist (LOR3A) was spin coated at 4000 rpm and baked on a hotplate at 180 °C for 5 min. A second layer of S1813 was spin coated at 4000 rpm and baked on a hotplate at 120 °C for 2 min. The pattern was written by a 60 mW laser beam after which the sample was developed in developer MF-318 for 50 s. The nanohole array was then prepared by colloidal lithography.[3] Finally, lift-off was performed in remover mr-Rem 400.

Desorption of proteins: The surfaces were immersed in a solution of proteins for 30 min to ensure saturated binding, using the kinetics from SPR and QCM measurements as guideline. For protein loading by hydrogen bonds, the surface was rinsed in PBS pH 5.0 and water, then dried with N_2. Desorption was performed by immersing the samples in serum at its native pH. Alternatively, electrochemical release was carried out in a beaker, with a Pt cage as counter electrode and a Ag wire as reference electrode. The wire was preconditioned by depositing chloride ions by applying a +0.5 V (vs the Pt counter) for 10 min in 1 M HCl.

Protein conjugation: Two different fluorophores were used in the conjugation of fluorescent dyes to the amines of BSA: Alexa Fluor 488 and 555 (with tetrafluorophenyl or N-hydroxysuccinimide ester groups). A BSA solution (100 µL, 10 mg/mL, pH 8.5) was mixed with Alexa Fluor dye (100 µg), and the resulting solution was inverted every 10 min for 1 h. The reaction was terminated by the addition of PBS pH 5.0, which reduced the pH to 5.0 and diluted the sample to a protein concentration of 0.2 mg/mL. The fluorescent proteins were immobilized in PMAA brushes in the same manner as the native proteins.

Fluorescence microscopy: All fluorescence measurements were conducted using a Zeiss Axio Observer 7 inverted microscope equipped with an Axiocam506 camera. Microelectrodes in air were imaged using a 10× objective. For Alexa Fluo 488, excitation was at 450-490 nm and emission was collected at 500-550 nm. For Alexa Fluo 555, excitation was at 533-558 nm and emission was collected at 570-640 nm.

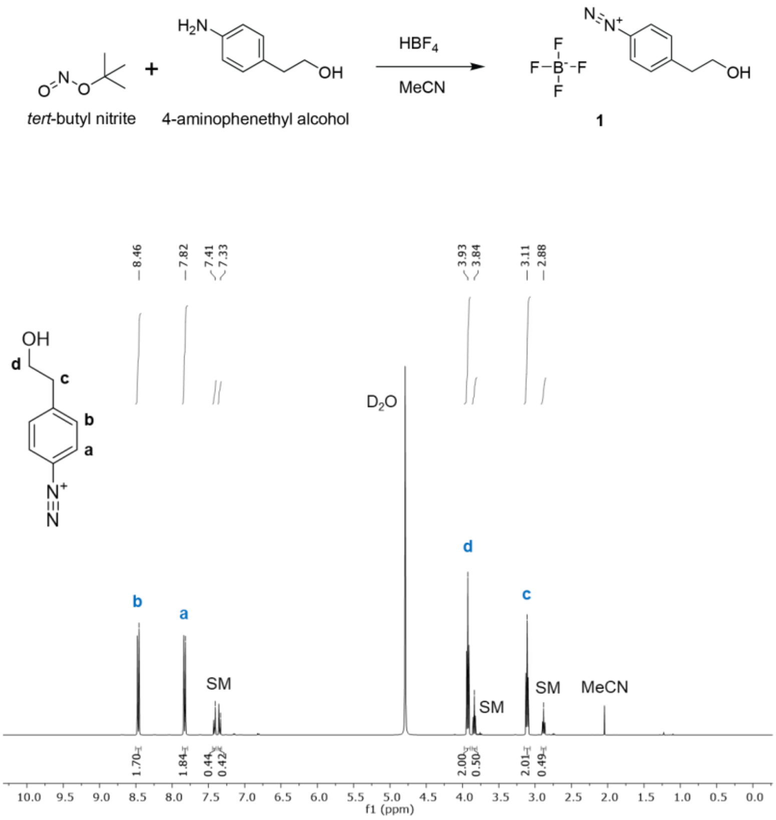

Figure S1. Synthesis of diazonium salt **1** and ^{1}H NMR (400 MHz, D_2O) spectrum of diazonium salt **1**, where SM represents the starting material 4-aminophenethyl alcohol.

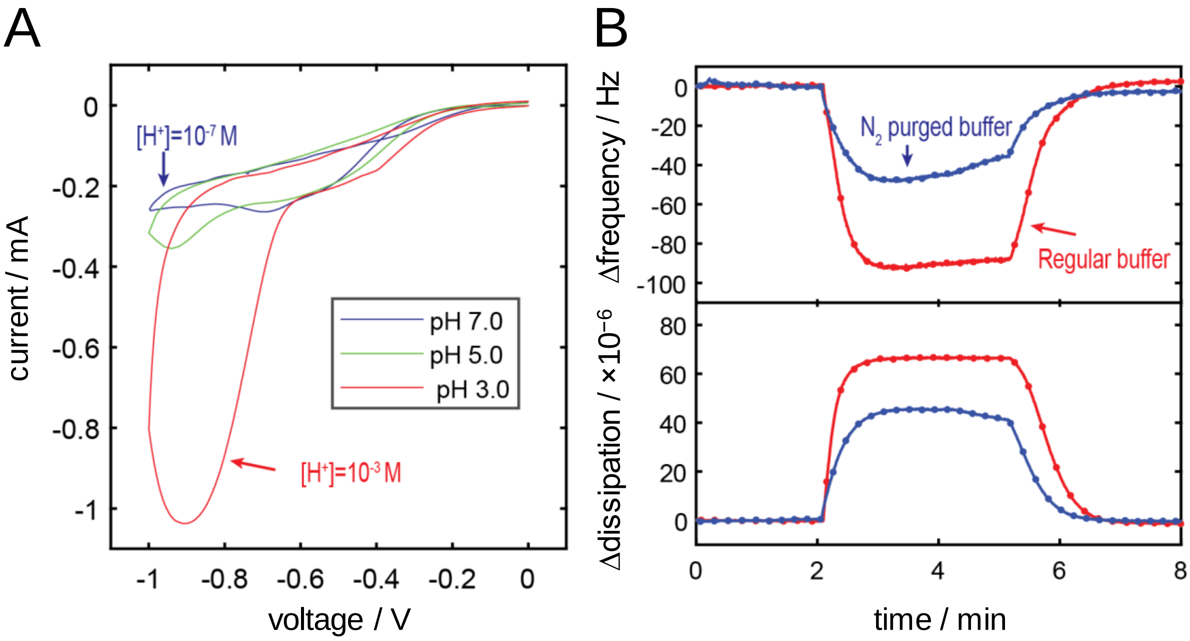

Figure S2. A) CV scans of PMAA brushes on QCMD Au sensors measured at different pH. A higher current for the negative sweep is obtained at lower pH, showing that protons contribute to the Faradaic reactions. B) QCM signals for a PMAA brush when an electrochemical potential is applied in a buffer with normal O_2 content (in equilibrium with ambient air) compared to an O_2 depleted buffer (purged with N_2). The brush clearly switches less after N_2 purge.

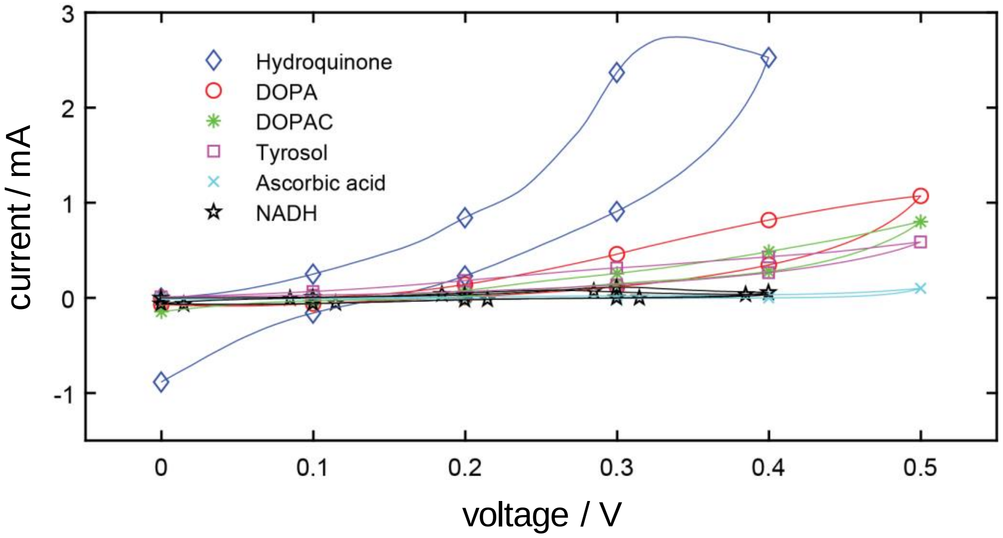

Figure S3. Cyclic voltammetry of gold electrodes for different reducing agents. The concentration of the species was 5 mM (in PBS pH 7.4) in each case. Faradaic currents are observed in most cases. For DOPA, the redox activity is quite strong initially but quickly decreases, most likely because an organic coating is formed due to electropolymerization. Several species are capable of acidification but hydroquinone is clearly the most efficient.

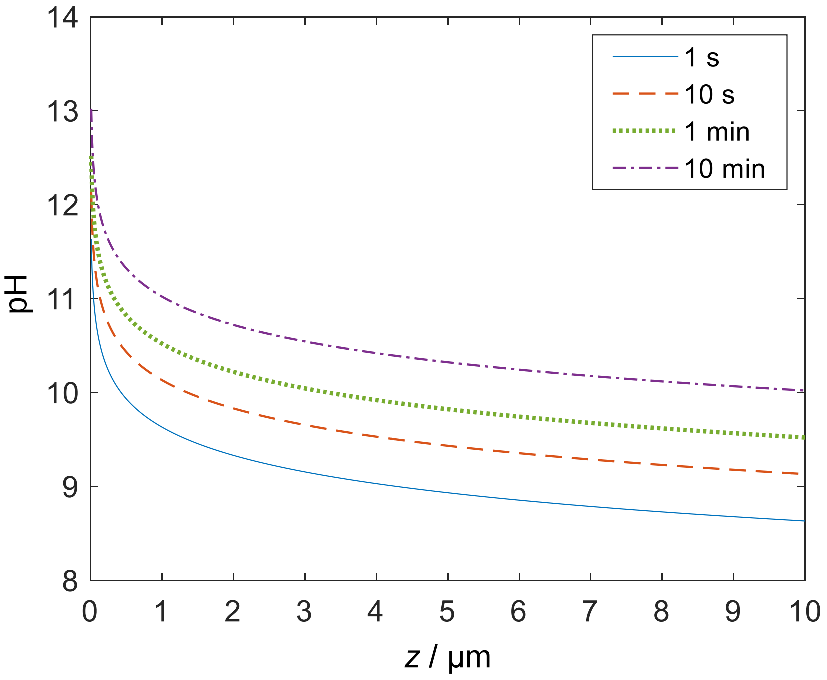

Figure S4. Calculated local pH as a function of distance *z* from the surface after different pulse times. The case of pH increase by O_2 reduction is modelled. The bulk pH is 7.4 and the diffusivity of protons is set to 9.3×10^{-9} m^2/s. Already after ~1 s, there is a noticeable pH change even z = 10 μm from the surface due to the very high diffusivity of protons. In the brush region ($z \approx 100$ nm) the pH goes up to ~12. This explains the efficient switching and that electrostatic repulsion (pH > pI) can be achieved for all proteins. For pH lowering, we found no analytical expression, but the local pH should be on the order of $\log_{10}(2/C_s)$ where C_s is the concentration of the proton producing species in units of M. (See the derivation at the end of this document for details.)

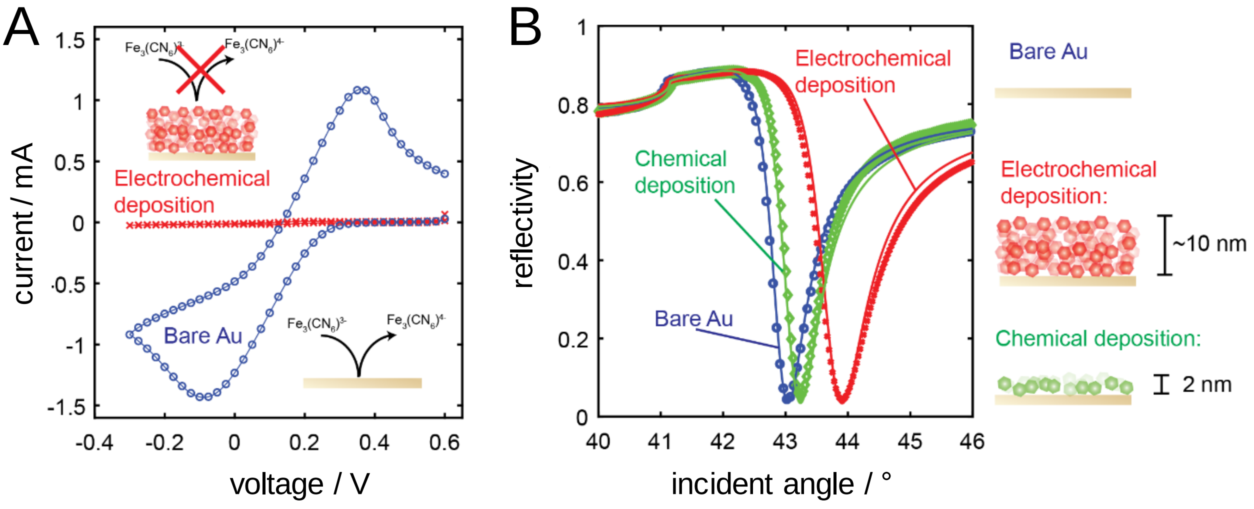

Figure S5. Electrochemical attachment of diazonium salts does not produce an interface capable of Faradaic reactions. A) CV scans in the presence of 5 mM ferrocyanide before and after electrografting, showing no current after layer formation. B) SPR scans in air comparing the chemical attachment presented in this work with electrografting. For electrografting, a single potential sweep was performed from 0 V to −1 V at 100 mV/s. The electrografted layer is clearly much thicker (values from analysis of the SPR spectra) and it is not surprising that it prevents redox reactions.

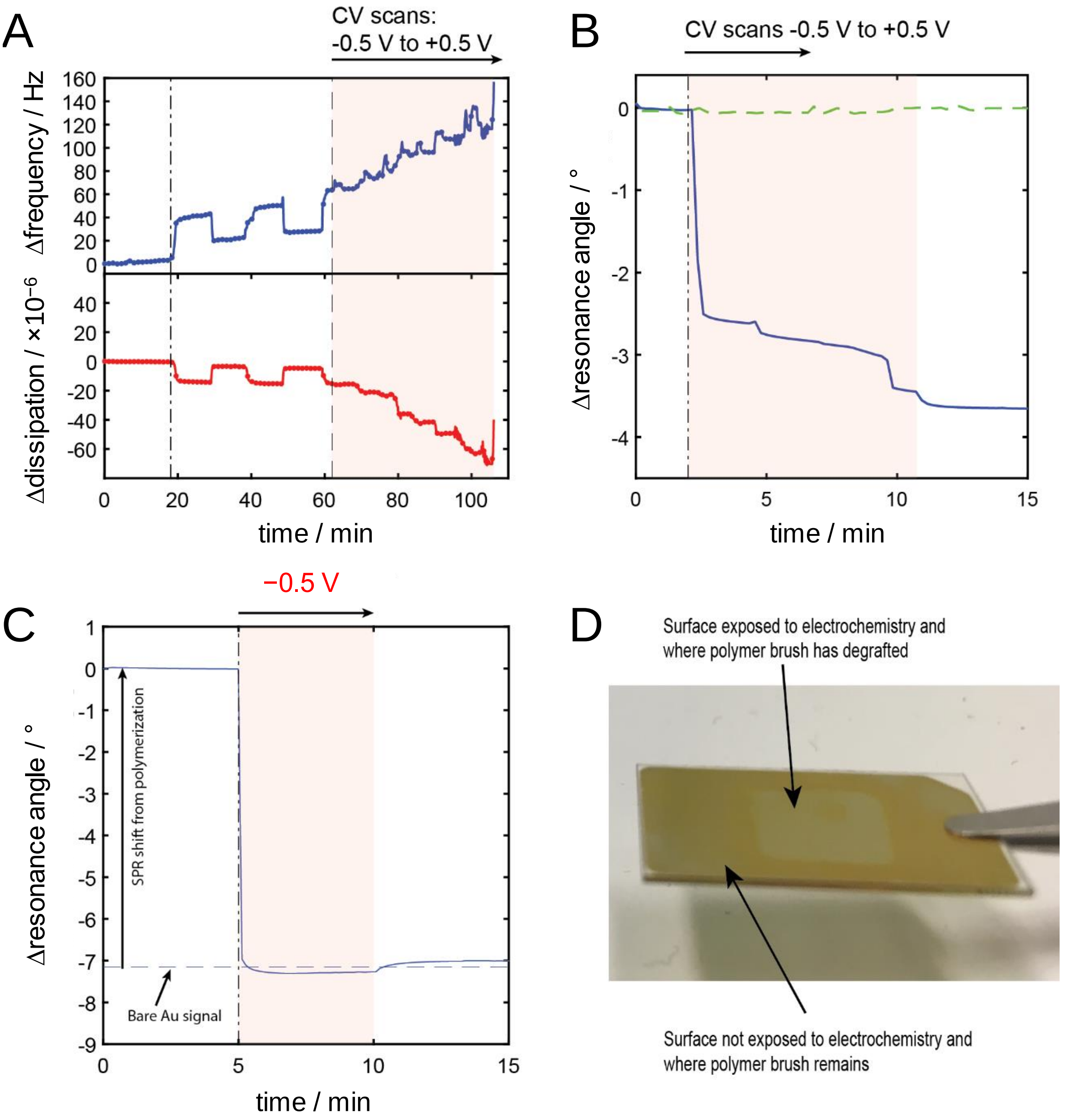

Figure S6. Demonstration of instability of thiol linkers to gold electrodes upon applying electrochemical potentials. A) QCMD measurement where initially a thiol-grafted brush is switched twice by changing the pH with different buffers, followed by attempts to non-destructively perform CV sweeps within the potential range −0.5 to +0.5 V vs Ag/AgCl (shaded region). Material clearly leaves the surface. B) Electrochemical SPR measurements where the same trend is observed. The time at which the electrochemical signal is applied is indicated and the corresponding SPR signal of a bare Au sensor is shown (dashed). C) Electrochemical SPR experiment where a constant potential of −0.5 V is applied for 5 min. Total desorption of the brush is observed within seconds, as confirmed by a control Au surface. D) Photo of the SPR sensor surface showing that the thiol-anchored polyelectrolyte brush is visibly removed.

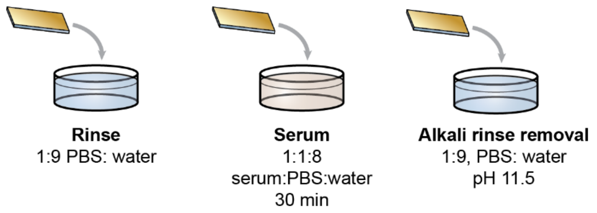

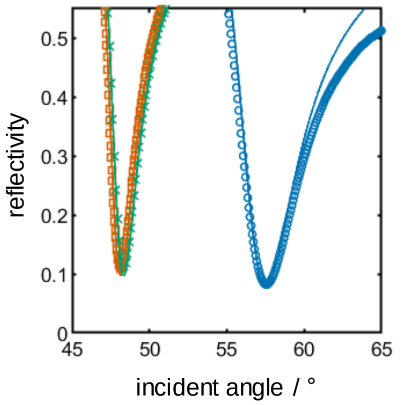

Step	Thickness (nm)
Initial	32.0 ± 0.2
Serum	64.6 ± 1.1
Alkali rinse	35.6 ± 2.5

Figure S7. Complementary SPR measurements in dry state after exposing surfaces to serum (see further in main text), but removing proteins by rinsing in pH 11.5 instead of electrochemistry. The very small remaining resonance shift (0.2°) in the fitted Fresnel model is practically identical (<0.1° difference) to the remaining shift after electrochemical release (main text). Furthermore, both values are within the error margin of repeated scans if the sample is removed and placed in the instrument again. The thickness in each step is that obtained when modelling a homogenous coating with refractive index 1.5 (brush and proteins).

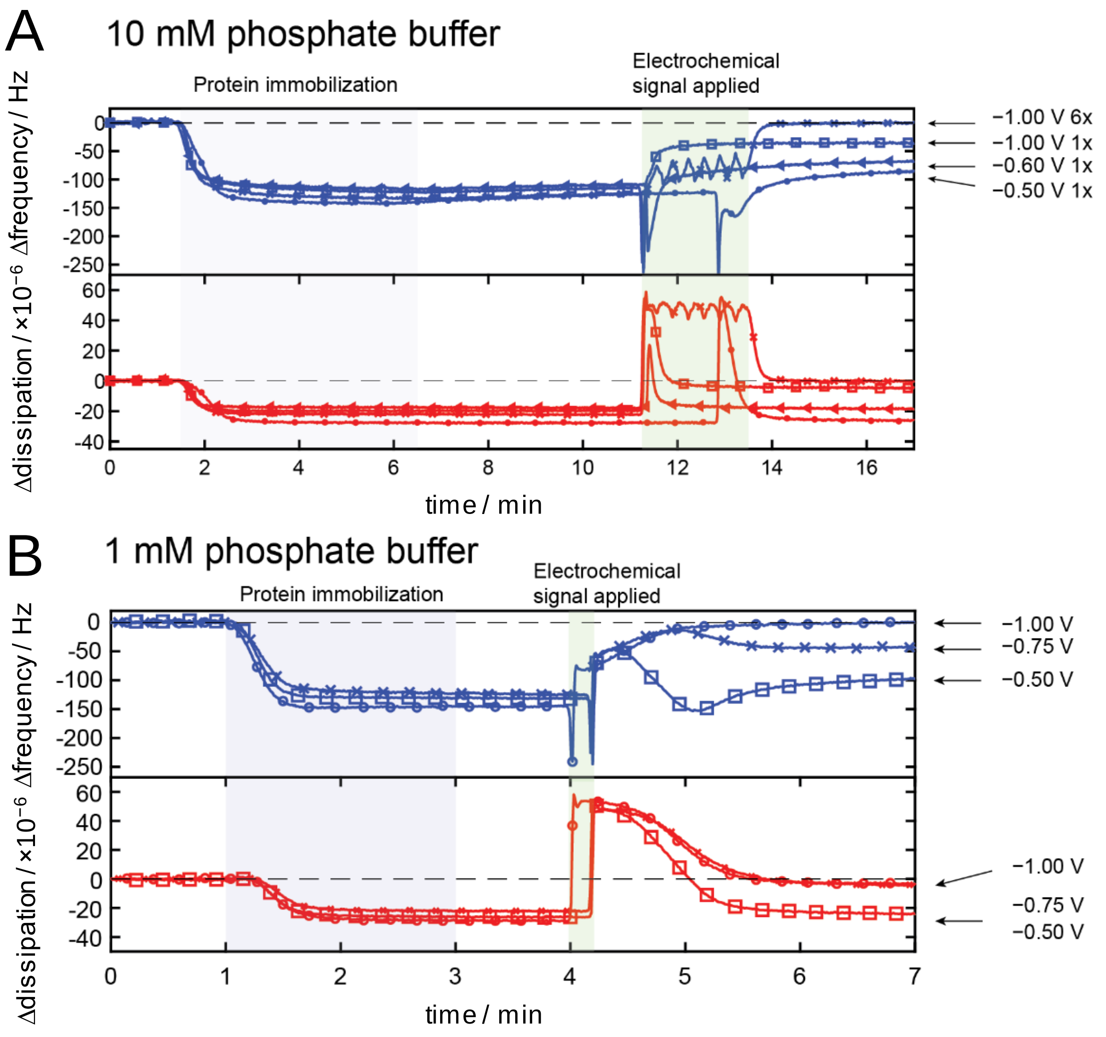

Figure S8. BSA immobilization followed by electrochemical desorption in PBS pH 5. A) 10 mM (standard) buffer strength. B) 1 mM buffer strength. Dashed horizontal lines represent the baseline. The end voltage and number of sweeps are indicated. For the higher buffering capacity in panel A, a higher potential magnitude and an increased number of cycles are required for full protein release.

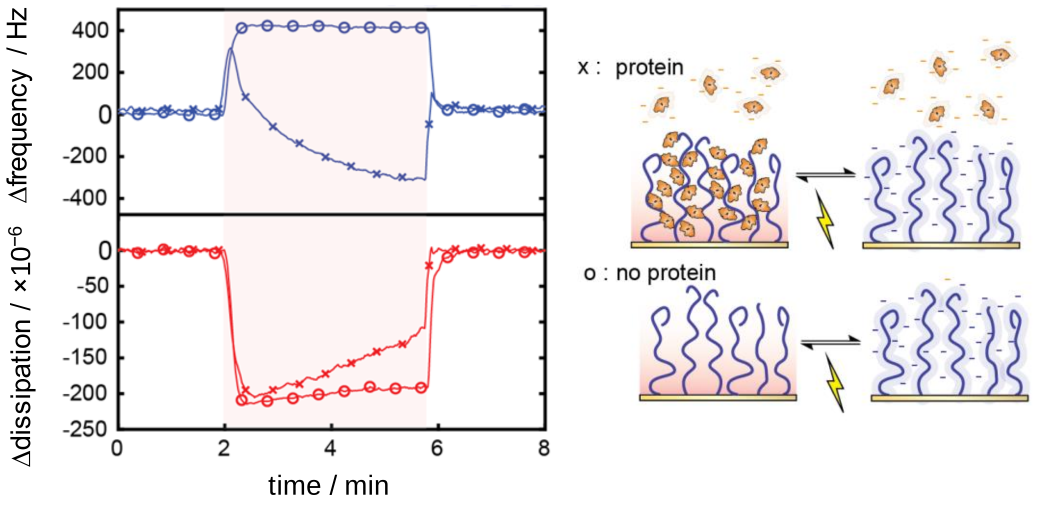

Figure S9. Example of protein binding on demand by acidification. Binding of BSA (circles) is induced by an oxidative potential (+0.5 V vs Ag/AgCl) that lowers the pH so that hydrogen bonds can be formed with protonated PMAA (pH_{bulk} = 7.4, 5 mM hydroquinone). Note that BSA is present throughout the whole measurement (crosses), i.e. only the potential is switched on/off. The control shows the QCMD response from the brush in the absence of BSA (circles).

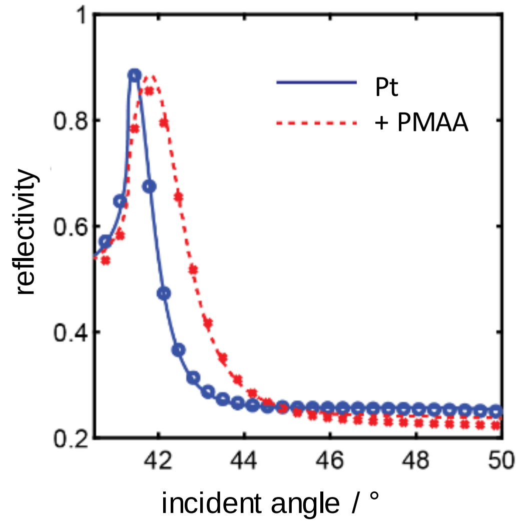

Material	Thickness (nm)	*n*	*k*
Air	Semi-infinite	1	0
Polymer	Fitted	1.5	0
Initiator	2	1.5	0
Pt	20	2.7	5.7
Cr	1	3.32	3.61
Glass	Semi-infinite	1.52	0

Figure S10. SPR data at 670 nm on 20 nm Pt, confirming brush synthesis after diazonium grafting and ATRP. The analysis is performed by fitting Fresnel models just like for Au but with different parameters for the metal permittivity. Although no clear resonance dip appears in the spectra, the region next to the total internal reflection shows intensity changes that are sufficient to determine surface coverage. In the initial “background” model, the polymer is excluded. When fitting the spectrum after polymerization, only the thickness is allowed to vary. For the particular spectrum in the plot, the fitted (dry) PMAA thickness was 15 nm.

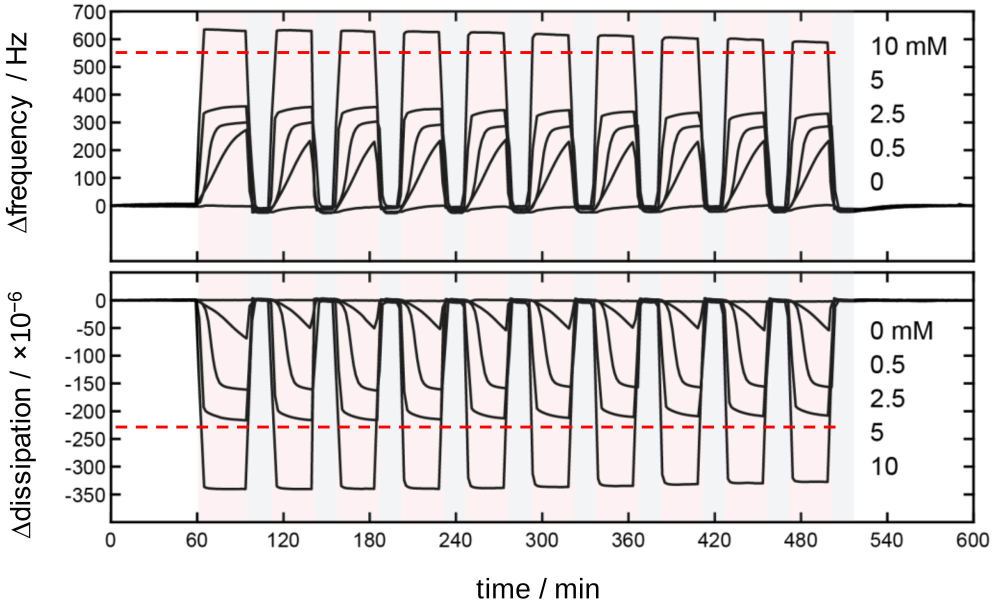

Figure S11. Supplementary electrochemical QCMD measurements of acidification on a Pt brush interface by H_2O_2 and anodic potentials. The brush gradually switches more to the protonated state when the H_2O_2 concentration increases. The dashed lines indicate the response to a bulk pH change to 5.0 for the same brush (running buffer is PBS pH 7.4). Note that there is barely any detectable signal from applying a potential in the absence of H_2O_2 (~10 Hz response).

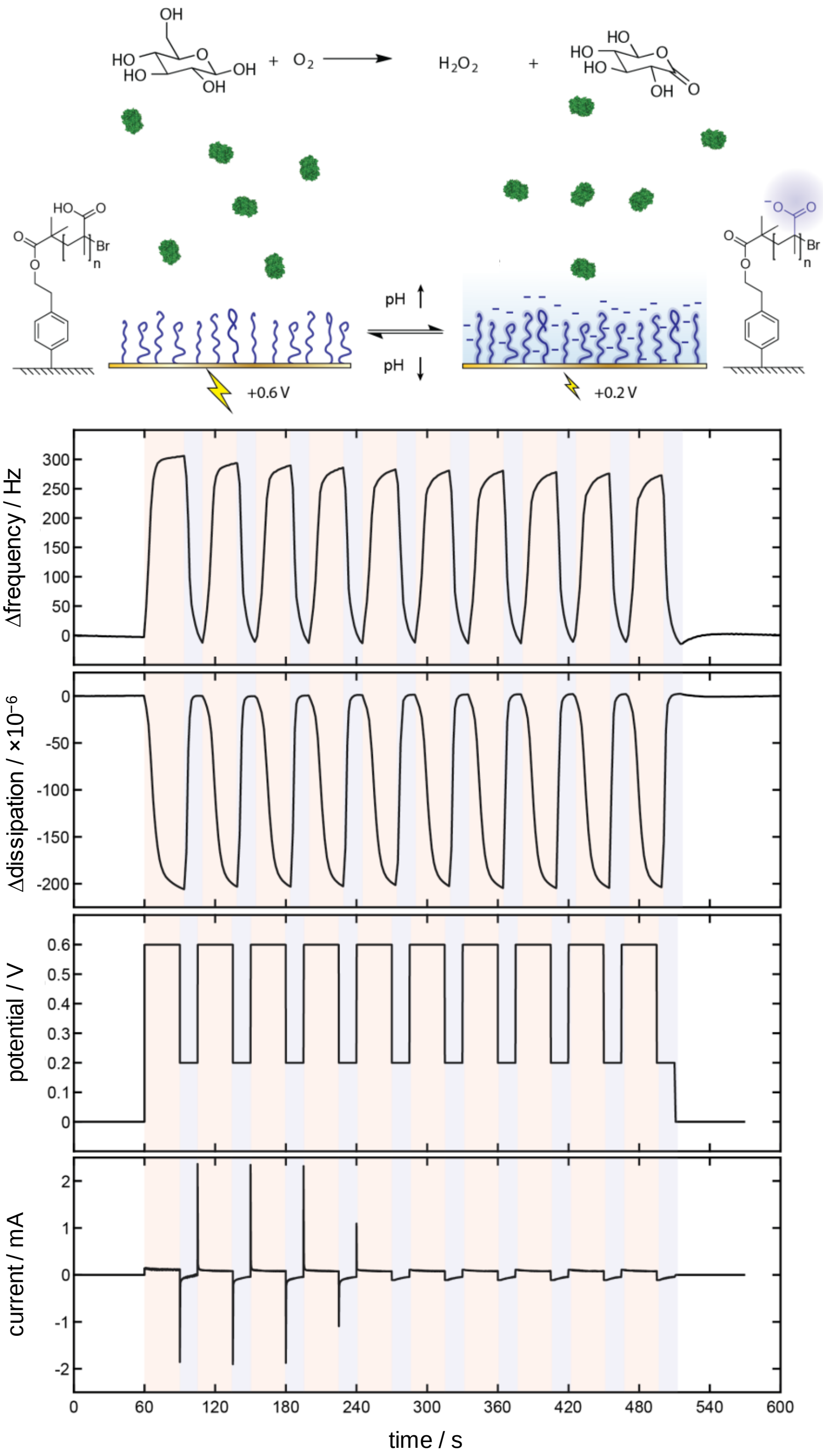

Figure S12. Supplementary electrochemical QCMD experiment of enzymatic acidification by glucose breakdown. The PMAA brush on Pt switches when glucose (10 mM) and GOX (60 nM) are present in solution (~300 Hz response, similar to the data in main text). Note that unlike in Figure S11, no H_2O_2 is added in solution as it is produced by GOX.

Table S1. Summary of results from electrochemical QCMD and SPR experiments of hydrogen bond immobilization and release of different proteins. In all cases, immobilization was performed at pH 5 by exposing the surface to a protein concentration of 0.3 mg/mL for ~10 min. The irreversible signals from immobilization (after rinsing) were on the order of ~100 Hz (QCMD) or a few degrees (SPR). Larger proteins tended to immobilize in higher amounts, while no correlation was seen with respect to isoelectric point. However, here the purpose is to look at the pH increase required for full release. The results show that proteins with high pI require a higher pH to leave the surface. The data set also indicates that smaller proteins are more easily released and many are fully desorbed even under conditions where their net charge is actually opposite to that of the brush. Finally, we note that almost no protein with net negative charge remains bound when the brush is negatively charged.

Protein	*M* (kg/mol)	pI	Release
Fibrinogen	340	5.5	>80% release by −0.3 V for 2 min. Fully released at bulk pH between 8 and 11.
Avidin	66	10	No release by −0.3 V for 2 min. Fully released by 20 sweeps down to −1.0 V or at bulk pH ~10.
NeutrAvidin	60	6.3	>50% released by −0.3 V for 2 min. Fully released when bulk pH reached ~10.
Bovine serum albumin	67	5.4	>90% released at bulk pH 6.
Insulin	6	5.3	>70% released by −0.3 V for 2 min. Fully released by −1.0 V for 5 min.
Lysozyme	15	11	No release by −0.3 V for 2 min or at bulk pH 8.0. Fully released after 5 sweeps down to −1.0 V.
Horseradish peroxidase	44	9	Full release by −0.3 V for 2 min.
Ribonuclease A	14	9.6	>60% released at bulk pH 6. >90% released at bulk pH 7. Fully released at bulk pH 8.
Proteinase K	30	8.9	Fully released already at bulk pH 6.
α-chymotrypsin	25	8.8	>90% released at bulk pH 6. Fully released at bulk pH 7.
Lactoferrin	78	8.7	Fully released at bulk pH between 7 and 8.
IgG antibodies	~150	6.6-8.6	~90% released at bulk pH 6. Fully released at bulk pH 7.

Theory

Here we give a motivation why it is reasonable that the interfacial pH changes occur sufficiently fast and within a sufficiently thick layer to make the brush switch as observed in the experiments. Further details on the theory of voltammetry is available in extensive textbooks.[4] In the absence of convection, the concentration of a species at a given point in space and time is given by solving partial differential equations of mass transport (analogous to heat transport). We consider first the reduction of O_2 (pH increase by potential control).

Under the assumption that the potential is sufficiently high, the Faradaic reaction occurs immediately when the reactants are present at the electrode surface (diffusion control). Furthermore, the concentration of O_2 is normally 0.3 mM in aqueous solutions, which is much higher than that of protons at pH ≥ 5. Thus, we consider the mass transport of H^+ to be rate limiting. For a planar surface in contact with an infinite solution, the concentration profile is given (from heat transport analysis) as:

$$C(z,t) = \frac{1}{\sqrt{4\pi Dt}} \int_0^\infty \left[\exp\left(-\frac{[z-s]^2}{4Dt}\right) - \exp\left(-\frac{[z+s]^2}{4Dt}\right) \right] C_0 \mathrm{d}s \quad (1)$$

Here C_0 is the initial proton concentration in solution and $C = 10^{-\mathrm{pH}}$ (in M). For H^+ in water, $D = 9.3\times10^{-9}$ m^2/s. When C_0 is independent of z, the integral has a simple solution:

$$C(z,t) = C_0 \mathrm{erf}\left(\frac{z}{\sqrt{4Dt}}\right) \quad (2)$$

Here "erf" is the error function. It can be noted that the characteristic extension of the depletion zone is ~$[2Dt]^{1/2}$. Thus, even if a potential is applied only for 10 s, the depletion zone is over two orders of magnitude longer than the brush thickness (<1 μm). (The brush is not expected to have any major effect on proton diffusivity since it is hydrated.)

Expressed in pH units, Equation 2 becomes:

$$\mathrm{pH}(z,t) = \mathrm{pH}_0 - \log_{10}\left(\mathrm{erf}\left(\frac{z}{\sqrt{4Dt}}\right)\right) \quad (3)$$

Equation 3 is used to generate the plots in Figure S4.

If instead protons are produced by a redox active source (pH lowering), the differential equation is more complicated, at least when the diffusivity D_s of this molecule differs from that of protons. Indeed, this is the case, since protons diffuse exceptionally fast by the Grotthuss mechanism.[5] The analogous heat transport problem can be written by non-homogenous Neumann boundary conditions:

$$\frac{\partial C(z,t)}{\partial t} = D\frac{\partial^2 C(z,t)}{\partial z^2}, C(z,0) = C_0, \frac{\partial C(\infty,t)}{\partial z} = 0, \frac{\partial C(0,t)}{\partial z} = f(t) \quad (4)$$

The function $f(t)$ can be determined. Under diffusion control, the incident flux of molecules is known (as described for O_2 reduction) and this is what causes the outward flux of protons. By Fick's first law the boundary condition becomes:

$$f(t) = -\frac{nC_s}{D}\sqrt{\frac{D_s}{\pi t}} \quad (5)$$

Here C_s is the initial concentration of the "proton source" and n is the number of protons produced for every oxidation event. However, the partial differential equation can only be formulated, not easily solved. Still, to get an approximate value for the local pH, we can use the number of protons N that have been produced per electrode area A after a certain time:

$$\frac{N(t)}{A} = 2nC_s\sqrt{\frac{D_s t}{\pi}} \quad (6)$$

The characteristic thickness of the layer in which the protons have spread out by diffusion is ~$[2Dt]^{1/2}$ and the average proton concentration in this layer is, from Equation 6:

$$C_{\mathrm{surf}} = C_0 + \frac{2nC_s\sqrt{\frac{D_s t}{\pi}}}{\sqrt{2Dt}} = C_0 + nC_s\sqrt{\frac{2D_s}{\pi D}} \quad (7)$$

Notably, the time dependence disappears, i.e. the "interfacial pH" is constant, but the zone where the pH is changed grows in size indefinitely. Since C_0 is orders of magnitude lower than C_s in the experiments, we can approximate the local pH as:

$$\mathrm{pH}_{\mathrm{surf}} \approx \log_{10}\left(\frac{1.25}{nC_s}\sqrt{\frac{D}{D_s}}\right) \quad (8)$$

Note that C_s needs to be inserted with unit of M. Given that protons diffuse around 10 times faster than small molecules and n is typically 2 (hydroquinone and H_2O_2), we get $pH_{surf} \approx \log_{10}(2/C_s)$. Thus, from Equation 8 it is clear that a few mM concentration of the proton producing species could be sufficient to reach pH as low as ~3.

The main limitation in this model is that the buffering effect from weak acids and bases in the bulk solution is not accounted for. However, this is probably compensated by the fact that the brush extension from the surface is much less (at least an order of magnitude) than the characteristic diffusion distance. Although we present no exact function to describe the pH gradient during acidification, it must have a shape reasonably similar to that in Figure S4, but inverted. Therefore, if buffering species are ignored, the pH inside the brush will be even lower than the average value in the depletion zone. Buffering species will counteract this effect, but mostly in the outer region of the depletion zone (as they will also be depleted close to the surface). Therefore, Equation 8 should be valid for estimating the pH inside the brush.

Convection is very difficult to implement in this kind of analytical modelling, but it can be noted that flow generally reduces the extension of the interfacial region where the pH is altered.[6] This means that the buffering effect from the solution should become more noticeable. However, the flow will also deliver the molecules that cause the pH changes more efficiently to the electrode. These effects of the local pH should roughly cancel each other and thus it is not surprising that no effect of convection was observed in the experiments.

References

[1] S. Gam-Derouich, M. N. Nguyen, A. Madani, N. Maouche, P. Lang, C. Perruchot, M. M. Chehimi, *Surf. Interface Anal.* **2010**, *42*, 1050-1056.
[2] G. Ferrand-Drake del Castillo, G. Emilsson, A. Dahlin, *J. Phys. Chem. C* **2018**, *122*, 27516-27527.
[3] K. Xiong, G. Emilsson, A. B. Dahlin, *Analyst* **2016**, *141*, 3803-3810.
[4] R. G. Compton, E. Laborda, K. R. Ward, *Understanding voltammetry: simulation of electrode processes*, Imperial College Press, London, **2014**.
[5] N. Agmon, *Chem. Phys. Lett.* **1995**, *244*, 456-462.
[6] T. M. Squires, R. J. Messinger, S. R. Manalis, *Nat. Biotechnol.* **2008**, *26*, 417-426.

Author Contributions

G.F.D.dC. majority of experimental work, data analysis, writing of original draft

M.K. experimental work (polymer-protein interactions)

Z.A. experimental work (diazonium salt synthesis)

K.X. experimental work (microelectrodes)

R.L.N.H. experimental work (diazonium salt verification), supporting writing

A.D. supervision, data analysis, funding acquisition, project administration, final writing
